# Plasticized Polystyrene by Addition of -Diene Based Molecules for Defect-Less CVD Graphene Transfer

**DOI:** 10.3390/polym12081839

**Published:** 2020-08-17

**Authors:** Tuqeer Nasir, Bum Jun Kim, Muhammad Hassnain, Sang Hoon Lee, Byung Joo Jeong, Ik Jun Choi, Youngho Kim, Hak Ki Yu, Jae-Young Choi

**Affiliations:** 1 SKKU Advanced Institute of Nanotechnology (SAINT), Sungkyunkwan University, Suwon 16419, Korea; tuqeernasir166@gmail.com (T.N.); kbj454@gmail.com (B.J.K.); 2 School of Advanced Materials Science & Engineering, Sungkyunkwan University, Suwon 16419, Korea; hassnain.edu@gmail.com (M.H.); alfhdj@gmail.com (S.H.L.); jbj929@gmail.com (B.J.J.); cksoon16@gmail.com (I.J.C.); 3 Department of Materials Science and Engineering & Department of Energy Systems Research, Ajou University, Suwon 16499, Korea; kimyh9347@naver.com

**Keywords:** graphene transfer, polymer plasticization, flexible polymer film, polystyrene, polymer functionalization

## Abstract

Chemical vapor deposition of graphene on transition metals is the most favored method to get large scale homogenous graphene films to date. However, this method involves a very critical step of transferring as grown graphene to desired substrates. A sacrificial polymer film is used to provide mechanical and structural support to graphene, as it is detached from underlying metal substrate, but, the residue and cracks of the polymer film after the transfer process affects the properties of the graphene. Herein, a simple mixture of polystyrene and low weight plasticizing molecules is reported as a suitable candidate to be used as polymer support layer for transfer of graphene synthesized by chemical vapor deposition (CVD). This combination primarily improves the flexibility of the polystyrene to prevent cracking during the transfer process. In addition, the polymer removal solvent can easily penetrate between the softener molecules, so that the polymer film can be easily dissolved after transfer of graphene, thereby leaving no residue. This facile method can be used freely for the large-scale transfer of 2D materials.

## 1. Introduction

Since the realization of graphene flakes [1], a lot of research has been carried out due to its exceptional electrical and physical properties [2,3]. Several top down and bottom up approaches have been explored for high quality graphene synthesis by various research groups [4,5,6]. Among these technologies, the chemical vapor deposition (CVD) method has caught the attention of researchers due to its advantages in synthesizing large scale and high-quality graphene, using a transition metal substrate as catalyst [7,8,9,10,11,12,13]. This method, however, required an additional process of transferring the synthesized graphene from metal substrate to target substrates like SiO_2_ for device fabrications [14,15,16]. Among several transfer techniques, poly (methyl methacrylate) (PMMA) assisted graphene transfer method [17] has been adopted widely, due to simplicity process and strong adhesion between graphene and PMMA [18]. However, the strong adhesion can cause permanent bonding between PMMA and graphene layer, due to the presence of active bonding sites in PMMA molecule chain like hydroxyl and carboxyl links, which can attach to graphene edges and cannot be removed during subsequent dissolution step of removing this polymer layer after transfer [19,20,21,22,23]. The other main issue regarding PMMA assisted graphene transfer is related to intrinsic mechanical properties of the support layer polymer itself, since it must provide enough mechanical support and flexibility to graphene layer during its transfer. Otherwise, it can cause mechanical defects like cracks and holes in transferred graphene, due to poor flexibility and hardness values of polymer support layer (PMMA) itself. Recently, a number of new materials have been explored to avoid the residue forming issue found in PMMA assisted graphene transfer, which range from using water soluble polymers to paraffin [24,25,26,27]. To the best of our knowledge, mechanical properties of these support layer materials and their effect on quality transferred graphene have not been studied before.

To remove the complications regarding the wet transfer of graphene altogether, recently, we reported the use of polystyrene assisted graphene transfer with the addition of plasticizer named 4, 4′-Di-iso-propylbiphenyl (DIPB) [28]. Polystyrene was used to provide stable and residue free adhesion with underlying graphene, and DIPB is added to improve poor mechanical and physical properties of polystyrene itself. Resultant polymer support film demonstrated excellent properties of transferred graphene [28]. The large scale transfer of graphene using plasticized polystyrene showed viable method of roll to roll transfer of as-grown CVD graphene.

**Figure 1:**
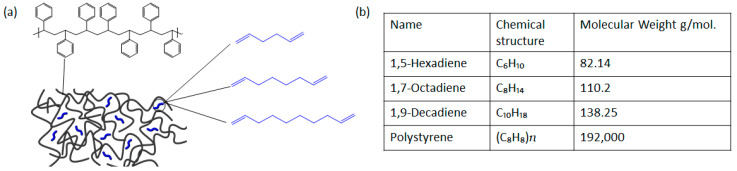
Polystyrene plasticizing and plasticizers. (**a**) Schematic of plasticized polystyrene (**b**) Chemical structure and details of polystyrene and plasticizers.

In this study, the effects of the length of plasticizer molecules mixed with polystyrene on the mechanical properties and flexibility were systematically tested and analyzed. It is intended to further develop previous published studies by applying to graphene transfer experiments. We have used polystyrene as base polymer for graphene transfer, due to the presence of multiple aromatic rings in polystyrene polymer chain, which can form strong π-π links with graphene layer for strong adhesion. Since polystyrene does not contain any active bonding links in its molecular structure, any unwanted and permanent bonding between the polymer and graphene can be avoided. Furthermore, polystyrene is readily soluble in wide array of organic solvents, leaving no residues after dissolution. Polystyrene has intrinsically amorphous structure and mechanical properties of polystyrene itself are not very suitable for graphene transfer, due to its rigid structure [29,30]. So, we introduced low weight plasticizer molecules during solution making of polystyrene to improve the mechanical flexibility of the support layer film when used for graphene transfer. Furthermore, 1-5 hexadiene, 1-7 octadiene and 1-9 decadiene molecules (similar molecular structure but has different length, See the Figure 1a of concentration of up to 20 w.t% were used for the plasticization of polystyrene. This simple plasticization process helps to provide the perfect combination of properties desired for an effective polymer layer to be used in graphene transfer, without any unwanted side effects of residues or mechanical defects. The results reported in this work demonstrate the possibility of this new material combination to be used for graphene transfer, both at lab and industry scale.

## 2. Materials and Methods

### 2.1. Preparation of Polymer Solution

Polymer solutions for graphene transfer were prepared by dissolving polystyrene beads (average MW ~192K by Sigma Aldrich, St. Louis, MO, USA) in toluene solvent at 5 w.t%. 1-5 hexadiene, 1-7 octadiene and 1-9 decadiene (by Sigma Aldrich, St. Louis, MO, USA) were used as plasticizers for polystyrene. Polystyrene to plasticizer ratio in the solution was varied from 100% pure polystyrene to 80/20 ratio of polystyrene with plasticizer. The solution was stirred at 500 rpm for 3 h and filtered to remove any undissolved residue from the solution. PMMA solution (4 wt% 950K MW PMMA A4 in Anisole by MICROCHEM, Westborough, MA, USA) was used for PMMA assisted graphene transfer.

### 2.2. Graphene Growth

Graphene was grown on copper foil (12.5 μm by Alfa Aesar, Haverhill, MA, USA) after cleaning the foil by nickel etchant (by Transene Co., Inc., Danvers, MA, USA). Low pressure CVD method was used to make full coverage graphene, as reported before [28]. Cleaned copper foils were loaded in a horizontal tube CVD system, and annealed at 1050 °C for 2 h at 100 sccm flow of hydrogen gas. Then, high purity methane gas (CH₄) was introduced in the presence of hydrogen gas for 10 min. Reaction chamber was cooled down to room temperature in under hydrogen gas flow before unloading the samples.

### 2.3. Graphene Transfer

As prepared polymer solution was spin coated on top of graphene layer at 500 rpm for polystyrene based solutions and 3000 rpm for PMMA solution for 30 s and dried in oven at 70 °C in oven for 10 min. This graphene layer, sandwiched between metal substrate and polymer support layer, was transferred on 300 nm SiO_2_/Si by wet transfer method [28] as shown in Supplementary Figure S1. After the transfer, this polymer support layer is removed conveniently by submerging the sample in toluene solvent for 30 min for polystyrene based polymer support layer, and in acetone for 30 min, in case of PMMA support layer, followed by washing off with acetone, ethanol and water spray.

### 2.4. Analysis and Measurements

Pure polystyrene and plasticized polystyrene films were investigated by FT-IR spectroscopy (Nicolet iS5 by Thermo Fisher Scientific, Waltham, MA, USA) at room temperature from 3500 to 500 cm^-1^, to detect any new bonding links between polystyrene and plasticizer materials. Mechanical properties are measured by nanoindentation test (Anton Parr instruments, Graz, Austria) at loading force from 0 to 30 µN. Surface morphology of transferred graphene was studied by optical microscope (KH-8700, HIROX, Tokyo, Japan), high resolution scanning electron microscope (JSM-6701 by JEOL, Akishima, Japan), at accelerating voltage of 10.0 kV, at a working distance of 7.2 mm and atomic force microscope (NX10 by Park Systems, Suwon, Korea). Raman spectra and mapping data were measured by Raman spectroscopy (UHTS-300, WITec, Ulm, Germany) at 532 nm laser wavelength and 1 µm spot size. Four-probe station was used to measure sheet resistance of transferred graphene (CMT-SR200N by AIT, Suwon, Korea).

## 3. Results and Discussion

**Figure 2:**
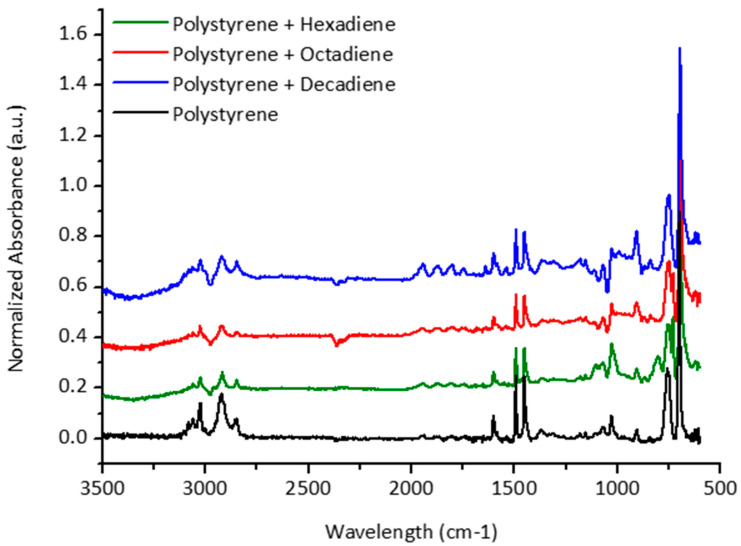
FTIR spectra of pure polystyrene and plasticized polystyrene.

The first step in our work was to prepare and identify a plasticized polystyrene material and its chemical and mechanical properties. The introduction of short chain molecules i.e., 1-5 hexadiene, 1-7 octadiene and 1-9 decadiene in polystyrene polymer structure can provide the necessary mechanical flexibility and improve the hardness of thin polymer film required for graphene transfer (Figure 1). A thin film of polystyrene and plasticized polystyrene was analyzed by FT-IR spectroscopy to identify peaks of all chemical bonds and detect any new bonds formed during the plasticizing of polystyrene. Figure 2 shows the normalized absorbance of pure polystyrene against a plasticized one. As we can note from the data, there is not any new peak generating from the plasticized polystyrene except the plasticizing agent itself, which shows that the materials are uniformly distributed and mixed without generating any unwanted bonding or links.

We also tested different molecular weight of polystyrene polymer and the effect of molecular weight on mechanical properties of polymer support film (Figure S2). It was observed that lowering the molecular weight resulted in higher film hardness values, which is not desired for supporting the polymer layer for graphene transfer.

**Figure 3:**
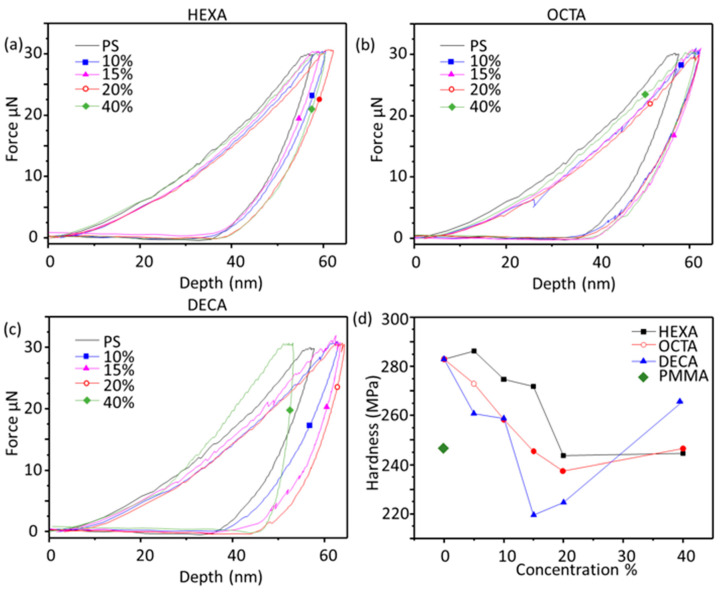
Nanoindentation and film hardness measurements for thin film. (**a**) Polystyrene + Hexadiene; (**b**) Polystyrene + Octadiene; (**c**) Polystyrene + Decadiene; (**d**) Comparison of Young’s modulus.

The main purpose of introducing plasticizer in polystyrene polymer is to increase the mechanical flexibility of the film, as pure polystyrene has an amorphous phase and high hardness value. These properties are studied by spin coating a thin film of polymer on SiO_2_ and measuring the film hardness (MPa) and tip penetration depth against a fixed force (30 µN in our case). Figure 3a,c shows load-displacement graph at different plasticizer concentrations; it can be noted that addition of plasticizer results in proportional increase in needle depth, which means film flexibility is increased due to the plasticizer. In addition, this plasticization effect of polystyrene film is clearly noted in film hardness graph (Figure 3d) due to the amount of plasticizer present in the polymer matrix. Lower film hardness values demonstrate the flexibility of the film and comparing to PMMA polystyrene film hardness is decreased as the plasticizer concentration is increasing, and it is clear that plasticizer can help to fine tune and control the mechanical properties of the polymer support film without the need of chemical modification of polymer itself. Plasticizer concentrations of up to 40% were tested, and it was observed that increasing the plasticizer ratio above resulted in decreasing film hardness, making it more flexible. However, the higher concentration of plasticizer resulted in hardness values increasing again, so we fixed the plasticizer ratio at 15%. Figure 3a,d demonstrate that increasing the chain length and molecular weight of plasticizer result in higher flexibility of polymer at the same plasticizer concentrations. Longer chain of plasticizer work to increase the spacing between polystyrene molecules, which increased the solubility and flexibility of the polymer support film.

**Figure 4:**
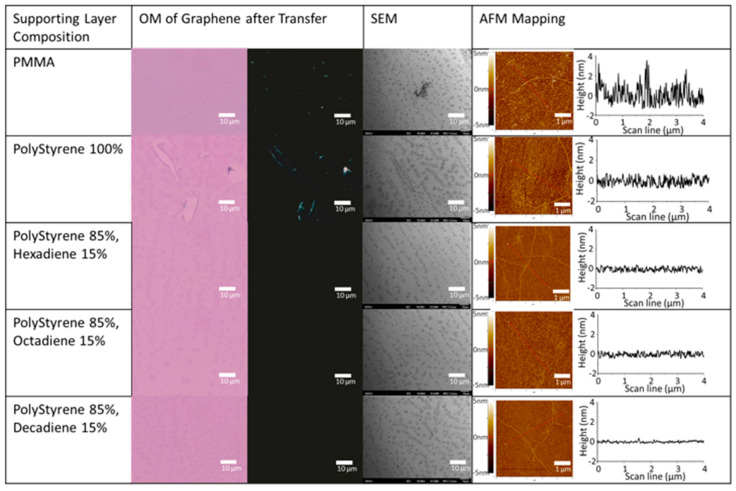
Comparison of Graphene quality after transfer (Bright field and dark field optical microscope, scanning electron microscope and AFM mapping images).

CVD grown graphene was transferred on ultrasonically cleaned SiO_2_ substrates, as described in the experimental section (Figure S1). After transferring graphene to 300 nm SiO_2_, the morphology of transferred graphene was analyzed in detail by optical microscope, scanning electron microscope and atomic force microscope (Figure 4). As expected, graphene transferred by PMMA is continuous but with polymer residue remains, which could not be removed by the solvents. It was observed that although pure polystyrene assisted graphene transfer resulted in residue free graphene, several cracks and tears in graphene sheet were detected due to the poor mechanical flexibility of the support layer polymer. After the addition of plasticizer in polystyrene matrix at ratio of 85%:15% polystyrene to plasticizer, optical microscope images show clean and continuous graphene film after transfer, without any cracks or tears in graphene film (Figure S3). Scanning electron microscope was used to obtain high resolution images of transferred graphene for detection of any residue particles and/or physical damage to graphene film. SEM images show that transferred graphene is defect free and continuous. To further investigate film morphology, samples were analyzed by atomic force microscope (AFM). PMMA assisted graphene showed very rough surface and multiple residue particles. AFM image of graphene transferred by polystyrene shows a residue free graphene, but with micro cracks and tears in graphene film, due to the poor mechanical flexibility of the polymer support film containing polystyrene only. Graphene transferred by plasticized polystyrene showed continuous and clean surface when analyzed by AFM. The results show the quality of graphene transferred by plasticized polystyrene to be very clean and the increased flexibility of plasticized polystyrene help to maintain the physical integrity of graphene without any cracks or tears due to sheering during the graphene transfer process.

**Figure 5:**
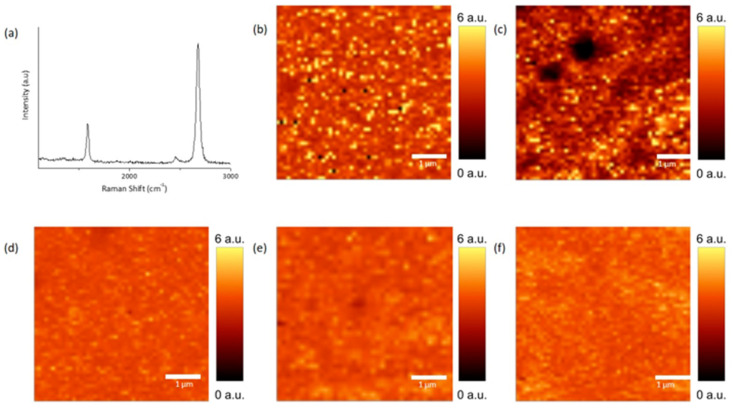
Raman spectra and mapping data after Graphene transfer. (**a**) Average Raman spectra of graphene showing predominately single layer graphene. (**b**,**f**) Raman mapping of 2D/G peaks after graphene transfer using (**b**) PMMA (**c**) Polystyrene (**d**) PS + Hexadiene (**e**) PS + Octadiene (**f**) PS + Decadiene.

Figure 5 shows the Raman spectra of graphene after the transfer process. Average Raman spectra of transferred graphene show predominantly single layer graphene with 2D/G ratio of >2 (Figure 5a). After the transfer, 2D/G peak mapping was carried out to visualize the film continuity and quality comparison, as shown in Figure 5b,f. Here it was clearly noticed that although the PMMA assisted graphene transfer resulted in continuous film, however, a lot of black spots were noticed in Raman mapping image, which relates to the residue particles detected in OM, SEM and AFM measurements earlier. Polymer support film of 100% polystyrene showed the residue free graphene properties, but the poor mechanical flexibility of the polystyrene resulted in tears/cracks in graphene film, as observed in Figure 5c. After the addition of plasticizer in polystyrene during the solution making step, the resulting combination showed excellent 2D/G peak mapping data shown in Figure 5d–f.

This result further supported the effectiveness of the plasticizing process of polystyrene. It was also observed that all three plasticizers (1-5 Hexadiene, 1-7 Octadiene, and 1-9 Decadiene) used for polystyrene plasticization process significantly improved the transfer quality of graphene, and retained the intrinsic properties and structure of primarily single layer graphene.

**Figure 6:**
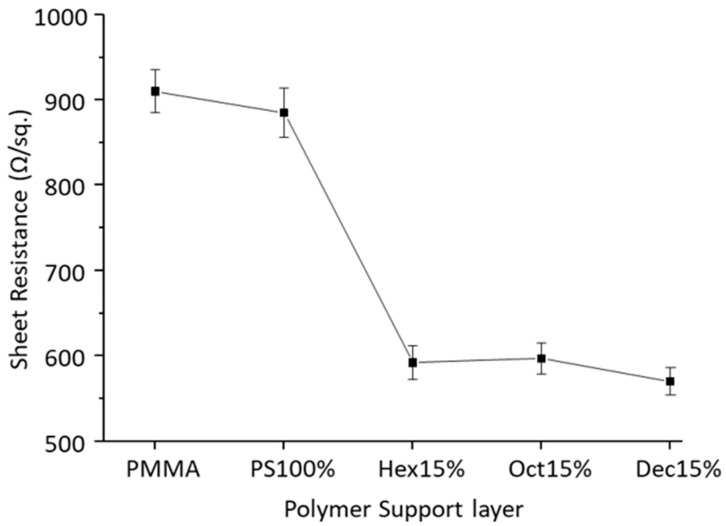
Average Sheet resistance measurement of transferred graphene.

Lastly, the electrical properties of these graphene samples were compared by measuring the sheet resistance (average of 5 readings in Ω/sq) of as transferred graphene samples. Figure 6 shows the average sheet resistance of graphene transferred by all the listed polymer support layers, and it can be noticed that the use of 100% polystyrene did not result in significant improvement over PMMA assisted graphene transfer due to the formation of cracks and tears.

The plasticized polystyrene however, resulted in much lower sheet resistance values compared to PMMA or polystyrene only, mainly due to the modified physical properties and flexibility of the polymer support layer. Lower sheet resistance values have been reported earlier for PMMA assisted graphene transfer, but that method required the additional process of the annealing of transferred samples, which added extra processing steps and costs [31].

## 4. Conclusions

In this work, we present a comprehensive study of the effect of mechanical and chemical properties of the polymer support layer used for graphene transfer, along with a brand-new material combination using polystyrene and low molecular weight softeners (hexadiene, 1-7 octadiene and 1-9 decadiene). This work demonstrates that flexibility and chemical reactivity of polymers used for graphene transfer play equal role to achieve the best quality of CVD graphene, and will help to overcome the major hurdle of degradation of graphene during the transfer process to desired substrates. Plasticized polystyrene contributes to uniform graphene transfer without sacrificing mechanical and chemical properties of graphene film, as indicated by improved morphology and electrical properties, along with the removal of surface defects and chemical residues attributed to current graphene transfer techniques, like PMMA assisted transfer. This new material combination is expected to be easily scalable to large scale transfer application as well as the transfer of other 2D materials like TMDCs, hBN and black phosphorus.

## Supplementary Materials

The following are available online at https://www.mdpi.com/2073-4360/12/8/1839/s1, Figure S1: Schematic of conventional graphene transfer method on SiO_2_ substrate, Figure S2: Nano-indenter test (**a**) and hardness comparison (**b**) for different Molecular Weight Polystyrene film, Figure S3: Bright and dark field optical microscope images of graphene transfer by different polymer support layers.
