# Supplementary material for: Plasticized Polystyrene by Addition of -Diene Based Molecules for Defect-Less CVD Graphene Transfer
Source: Polymers (Basel). 2020 Aug 17;12(8):1839. doi: 10.3390/polym12081839 (PMC7465162; doi:10.3390/polym12081839)
Supplement: Supplementary file 1 [file polymers-12-01839-s001.pdf]

Supplementary Informations

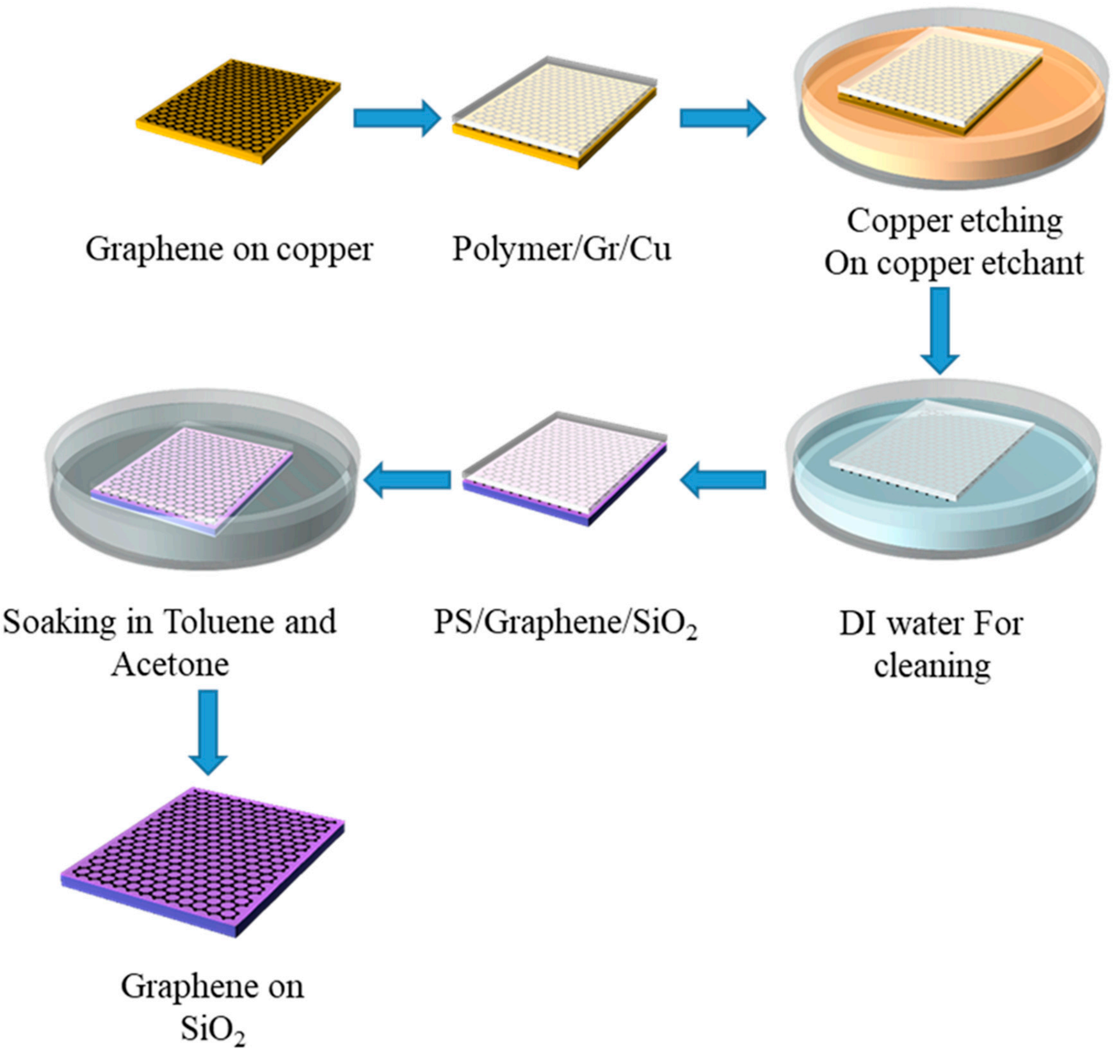

Figure S1. Schematic of conventional graphene transfer method on SiO_2 substrate.

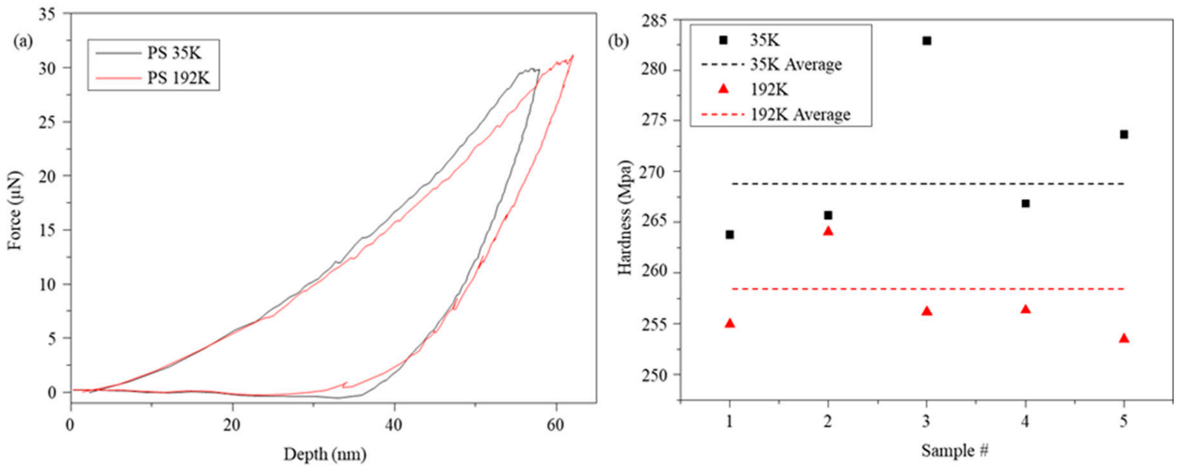

Figure S2. Nano-indenter test (**a**) and hardness comparison (**b**) for different Molecular Weight Polystyrene film.

Supporting Layer Composition	OM of Graphene after Transfer
PMMA	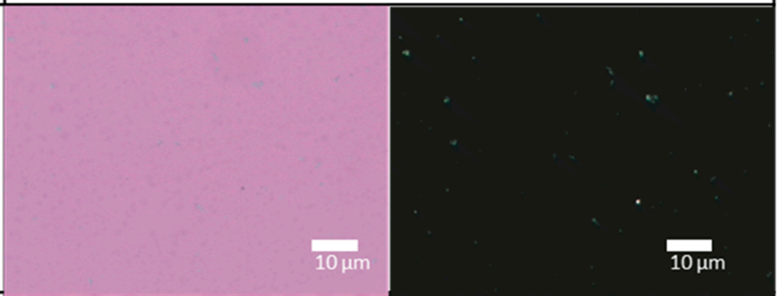
PolyStyrene 100%	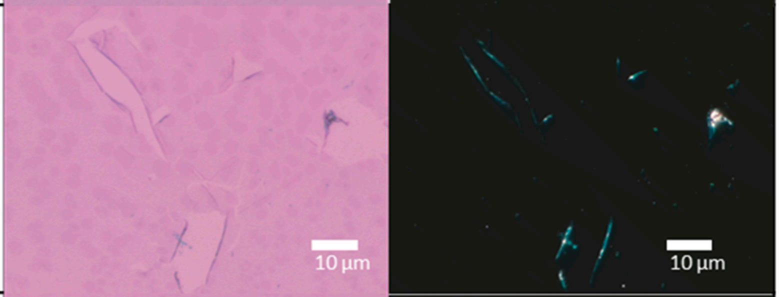
PolyStyrene 85%, Hexadiene 15%	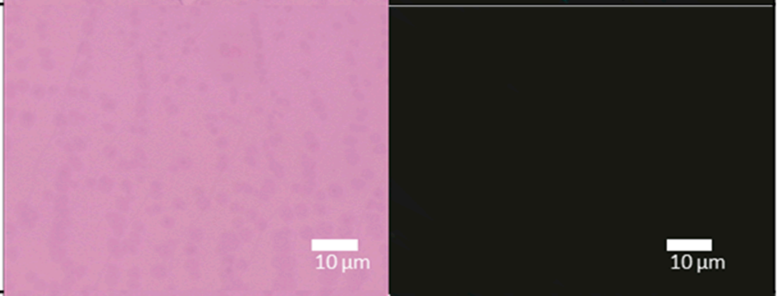
PolyStyrene 85%, Octadiene 15%	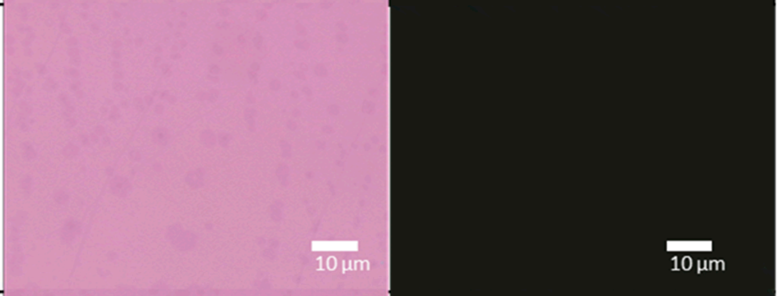
PolyStyrene 85%, Decadiene 15%	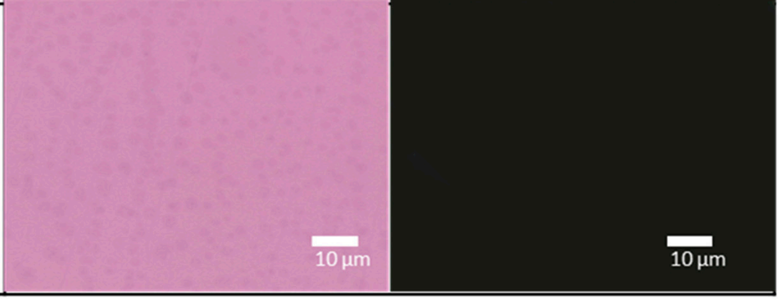

Figure S3. Bright and dark field optical microscope images of graphene transfer by different polymer support layers.
